# A New Insect-Specific Flavivirus from Northern Australia Suppresses Replication of West Nile Virus and Murray Valley Encephalitis Virus in Co-infected Mosquito Cells

**DOI:** 10.1371/journal.pone.0056534

**Published:** 2013-02-27

**Authors:** Jody Hobson-Peters, Alice Wei Yee Yam, Jennifer Wei Fei Lu, Yin Xiang Setoh, Fiona J. May, Nina Kurucz, Susan Walsh, Natalie A. Prow, Steven S. Davis, Richard Weir, Lorna Melville, Neville Hunt, Richard I. Webb, Bradley J. Blitvich, Peter Whelan, Roy A. Hall

**Affiliations:** 1 Australian Infectious Diseases Research Centre, School of Chemistry and Molecular Biosciences, The University of Queensland, St. Lucia, Queensland, Australia; 2 Australian Centre for Ecogenomics, The University of Queensland, St, Lucia, Queensland, Australia; 3 Centre for Disease Control, Health Protection Division, Northern Territory Department of Health, Darwin, Northern Territory, Australia; 4 Berrimah Veterinary Labs, Department of Primary Industries and Fisheries, Darwin, Northern Territory, Australia; 5 Centre for Microscopy and Microanalysis, The University of Queensland, St. Lucia, Queensland, Australia; 6 Department of Veterinary Microbiology and Preventative Medicine, College of Veterinary Medicine, Iowa State University, Ames, Iowa, United States of America; University of Texas Medical Branch, United States of America

## Abstract

Recent reports of a novel group of flaviviruses that replicate only in mosquitoes and appear to spread through insect populations via vertical transmission have emerged from around the globe. To date, there is no information on the presence or prevalence of these insect-specific flaviviruses (ISFs) in Australian mosquito species. To assess whether such viruses occur locally, we used reverse transcription-polymerase chain reaction (RT-PCR) and flavivirus universal primers that are specific to the NS5 gene to detect these viruses in mosquito pools collected from the Northern Territory. Of 94 pools of mosquitoes, 13 were RT-PCR positive, and of these, 6 flavivirus isolates were obtained by inoculation of mosquito cell culture. Sequence analysis of the NS5 gene revealed that these isolates are genetically and phylogenetically similar to ISFs reported from other parts of the world. The entire coding region of one isolate (designated 56) was sequenced and shown to have approximately 63.7% nucleotide identity and 66.6% amino acid identity with its closest known relative (Nakiwogo virus) indicating that the prototype Australian ISF represents a new species. All isolates were obtained from *Coquillettidia xanthogaster* mosquitoes. The new virus is tentatively named Palm Creek virus (PCV) after its place of isolation. We also demonstrated that prior infection of cultured mosquito cells with PCV suppressed subsequent replication of the medically significant West Nile and Murray Valley encephalitis viruses by 10–43 fold (1 to 1.63 log) at 48 hr post-infection, suggesting that superinfection exclusion can occur between ISFs and vertebrate-infecting flaviviruses despite their high level of genetic diversity. We also generated several monoclonal antibodies (mAbs) that are specific to the NS1 protein of PCV, and these represent the first ISF-specific mAbs reported to date.

## Introduction

Flaviviruses are responsible for a number of important mosquito-borne diseases of humans and animals in Australia, including dengue, Murray Valley encephalitis and Japanese encephalitis (JE) [1]. Dengue, JE, yellow fever and West Nile fever are also major medical problems around the world [2]. Flaviviruses are a group of small, enveloped viruses that contain a positive-sense RNA genome with a single open reading frame (ORF) which is flanked by 5′ and 3′ untranslated regions (UTRs). The ORF is translated as a single polyprotein, which is cleaved by viral and cellular proteases into three structural (C, prM and E) and seven non-structural proteins (NS1-NS5).

Flaviviruses are usually transmitted between arthropods and vertebrates and rely on replication in both of these hosts for their natural transmission cycle. In 1975, Stollar and Thomas reported the isolation of an unusual virus (cell fusing agent virus; CFAV) from mosquito cell cultures [3]. Further analysis revealed that CFAV is a distant relative of members of the flavivirus genus, but did not replicate in vertebrate cells. CFAV and similar viruses - Kamiti River virus (KRV) and Culex flavivirus (CxFV) - were subsequently isolated from mosquitoes in the wild and shown to belong to a distinct “insect-specific” flavivirus (ISF) lineage [4]–[6]. With the advent of improved molecular tools for viral detection, several new species of ISF including Aedes flavivirus (AeFV [7], [8]), Quang Binh virus (QBV [9]), Nakiwogo virus (NAKV [10]), Chaoyang virus (Genbank accession number FJ883471 – Wang et al., 2009), Lammi virus [11], Nounané virus [12], Calbertado virus [13] and Culex theileri flavivirus (CTFV [14]), have since been isolated from various regions of the world.

Data from several studies indicates that at least some ISFs are maintained in nature in the absence of a vertebrate host by vertical transmission from female mosquitoes to their progeny [15]–[17]. A lack of a direct association of these viruses with disease has largely seen ISFs ignored to date, however, recent reports by Kent et al. (2010) [18] and Bolling et al. (2012) [17] suggesting that co-infection with CxFV may enhance or suppress transmission of West Nile virus (WNV) in some vectors has created intense interest in the interaction of ISFs with other flaviviruses in mosquito cells. In this paper, we report the isolation and phylogenetic analysis of a new ISF detected in mosquitoes from northern Australia and the generation of ISF-specific recombinant proteins and monoclonal antibodies. We also provide *in vitro* evidence of “super-infection exclusion” of heterologous flaviviruses in cell cultures previously infected with this new virus.

## Materials and Methods

### Ethics Statement

The mouse work in this study was carried out under conditions approved by The University of Queensland Animal Ethics Committee (Animal Ethics Number 299/10). Surgery was performed under ketamine/Xylazine and all efforts were made to minimize suffering.

No specific permits were required for the described field studies and no specific permissions were required for the locations/activities for mosquito trapping because they are public lands and are not privately owned or protected in any way. The sites of mosquito trapping are those where the Northern Territory Department of Health conducts regular mosquito monitoring and has done so for many years. These field studies did not involve endangered or protected species.

### Trapping and Processing of Mosquitoes

Mosquitoes were trapped in March to June, 2010 from various sites around the Northern Territory of Australia. These sites included Darwin, Katherine, Alice Springs, Alyangula, Groote Eylandt, Jabiru and the McArthur River Mine. The mosquitoes were trapped by E.V.S. dry ice baited light traps [19], sorted to species in pools of 1 to 50 as previously described [20] and stored at −80°C until processed. All of the mosquitoes were female. Mosquito pools were homogenized for RNA extraction and virus isolation as previously described [21].

### Viral RNA Detection and Isolation from Mosquito Homogenates

Mosquito homogenates were screened for the presence of flaviviral RNA using the pan-flavivirus specific primers FU2 and cFD3 [22] using the methods described in Blitvich et al. (2009) [23]. Briefly, viral RNA was extracted from 200 µl of mosquito homogenate using the Qiagen RNeasy extraction kit as per the manufacturer's instructions and purified RNA was eluted in 50 µl of nuclease-free water. Five µl of purified RNA was then tested by RT-PCR (SuperScript III One-Step RT-PCR System with Platinum Taq DNA polymerase, Invitrogen) for the presence of flavivirus RNA. A second aliquot of mosquito homogenate from PCR-positive pools was inoculated onto monolayers of *Aedes albopictus* (C6/36) cells and incubated at 30°C for 7 days. Two hundred µl of culture supernatant was collected from the inoculated cultures and then total RNA was extracted and tested by RT-PCR as described above. Aliquots of culture supernatant from PCR-positive cultures were then stored at −80°C for further analysis.

### Sequencing of Viral Isolates

Initial sequencing of part of the NS5 genes for each viral isolate was performed on the RT-PCR product generated from the primer pair FU2 and cFD3 [22] using the protocol described above. The nucleotide sequence of the entire coding region of one PCV isolate (designated isolated 56) was determined by a combination of gene walking using a series of primers designed from PCV-derived nucleotide sequence and from regions of conserved sequence of published ISF genomes (NAKV, QBV, CxFV) and either a two-step RT-PCR using Superscript III reverse transcriptase (Invitrogen) and Phusion high fidelity DNA polymerase (Finnzymes) or the SuperScript III One-Step RT-PCR System with Platinum Taq DNA polymerase (Invitrogen). The 5′ end of the sequence was amplified using the GeneRacer kit (Invitrogen) according to the manufacturer's instructions The amplicons were purified by agarose gel electrophoresis and extracted using the NucleoSpin® Gel and PCR Clean-up kit (Macherey Nagel). The purified DNA fragments were sequenced at the Australian Genome Research Facility (Brisbane, Queensland). For some regions of the genome, a cloning passage was performed and sequencing performed directly on purified plasmid.

### Sequence Alignments and Phylogenetics

Virus sequences were aligned using the AlignX component of the Vector NTI suite (Invitrogen), and then inspected and edited using BioEdit [24]. Nucleotide and amino acid identities were calculated with BioEdit. Phylogenetic trees were constructed using the Phylip group of programs (neighbour joining trees) [25] and PhyML (maximum likelihood trees) [26]. Bootstrapped maximum likelihood trees were constructed using the NS5/3′UTR and complete open reading frames (ORF) of a selection of ISFs. The trees were mid-point rooted and are based on 1000 replicates. Specific parameters are available from the authors on request.

### Cell and Virus Culture

C6/36 cells (ATCC CRL-1660) were cultured in RPMI 1640 with 5–10% fetal bovine serum (FBS) and incubated at 28°C. The mammalian cells, African Green Monkey Kidney (Vero; ATCC CCL-81), baby hamster kidney (BHK-21; ATCC CCL-10), porcine stable equine kidney (PS-EK; [27]) and human adeno carcinoma (SW-13 CCL-105) were cultured in Dulbecco's modified Eagle's medium (DMEM) containing 2% FBS, while the hybridoma cell lines produced in this study were grown in hybridoma serum free medium (Invitrogen) initially supplemented with 20% FBS and then weaned to serum-free culture for antibody production. COS-7L cells (Invitrogen) were maintained in RPMI 1640 with 2% FBS. All mammalian cells were incubated at 37°C with 5% CO_2_. All media were supplemented with 50 U penicillin/mL, 50 µg streptomycin/mL and 2 mM L-glutamine.

Each virus isolate was propagated by inoculating onto monolayers of C6/36 cells and incubation at 28°C for 5–7 days before harvesting. The viral titre was determined by 50% tissue culture infective dose (TCID_50_) assays using methods described by May *et al.* (2006) [28].

To enhance the visualisation of cytopathic effect (CPE), the growth medium on PCV-infected C6/36 monolayers was replaced with medium that had been adjusted to pH 6.0 using sterile-filtered 1M MES (2-(N-Morpholino)ethanesulfonic acid hydrate) buffer. Images of the monolayers were captured 24 hr after changing the medium.

### Analysis of Virus Replication in Mammalian Cell Lines

Stocks of PCV (isolate 56) and WNV (Kunjin virus MRM16; WNV_KUNV_), prepared in C6/36 cells, were used to inoculate a panel of mammalian cell lines commonly used in the culture of flaviviruses. These included Vero, BHK-21, PS-EK and SW-13 cells. After culturing for 7 days, the inoculated cells were examined for the presence of CPE and cell monolayers were tested for viral RNA by RT-PCR using the FU2/cFD3 primer pair.

### Morphology of Viral Particles under Transmission Electron Microscopy (TEM)

PCV particles from C6/36 cell culture supernatant collected at 5 days post-innoculation were concentrated through a high molecular weight cut-off (300 K) centrifugal concentrator (Sartorius). Concentrated samples were placed onto a formvar-coated copper grid, negatively stained with 1% uranyl acetate and viewed in a JEOL1010 transmission electron microscope.

### Production and Characterisation of Monoclonal Antibodies to Palm Creek Virus

Hybridomas were generated to PCV proteins by immunizing an adult female BALB/c mouse with partially purified viral antigen. Briefly, PCV particles from PCV-infected C6/36 cell culture supernatants were concentrated through a high molecular weight cut-off (300 K) filter or by ultracentrifugation at 28,000 rpm (SW41Ti rotor Beckman) for 4 hr at 4°C. Aliquots of these preparations, along with Titre-Max Gold adjuvant (Sigma-Aldrich) diluted according to the manufacturer's instructions were used to immunize the mouse subcutaneously with three doses given at 14–28 day intervals. The mouse was boosted with a fresh preparation of concentrated PCV viral antigen (no adjuvant) about five months later and the spleen harvested three days following this. Fusion of the spleen cells with myeloma cells was performed as previously described [29]. Hybridomas secreting antibodies reactive to PCV-infected C6/36 cells were identified by enzyme-linked immunosorbent assay (ELISA) using previously described methods [30]. However, in this case, incubation steps were performed at 37°C to enhance sensitivity of the assay. The hybridomas were cloned by limit dilution and harvested culture supernatant stored under sterile conditions at 4°C until used.

PCV-reactive monoclonal antibodies (mAbs), were further tested for reactivity in immunoflourescent antibody assay (IFA), Western blot and fixed-cell ELISA. The reactivity of the anti-PCV mAbs to PCV viral lysates in Western blot was performed using previously published methods [30]. Each mAb was assessed for viral specificity by testing against a panel of vertebrate-infecting flaviviruses as described previously, except that an incubation temperature of 37°C was used [30], [31]. The isotype of each mAb was determined using the Mouse typer kit (Bio-Rad) according to the manufacturer's instructions.

### Immunofluorescence Assay (IFA)

C6/36 cells were grown on glass coverslips and infected with PCV or WNV_KUNV_ at a multiplicity of infection (M.O.I.) of 0.1, or sham-infected for 5 days, fixed in acetone and then blocked with 0.2% bovine serum albumim (BSA) in phosphate buffered saline (PBS) for 1 hr. Following incubation with the relevant mAbs in hybridoma cell culture fluid, the coverslips were washed with PBS and then stained with Alexafluor 488-conjugated goat anti-mouse IgG (H+L) (Invitrogen) and Hoechst 33342 nuclear stain (Invitrogen) for 1 hr or 5 min at room temperature respectively. Following another 3 washes in PBS, the coverslips were mounted onto glass microscope slides using ProLong Gold Anti-fade (Invitrogen) and viewed under the ZEISS LSM 510 META confocal microscope.

### Expression of Recombinant PCV prM/E, NS1 and NS5 Proteins in Mammalian Cells

The genes for the PCV pre-membrane (prM) and envelope (E) (minus the predicted sequence for the transmembrane domain), NS1 and NS5 (lacking the 5′ coding sequences for the first 85 amino acids of the predicted protein for NS5) proteins were amplified by high fidelity RT-PCR and cloned into the mammalian expression vector pcDNA3.1+ (Invitrogen) to generate three separate constructs – one each for prM/E, NS1 and NS5. For each, the 3′ end of the PCV gene was fused to the V5 epitope sequence followed by a polyhistidine sequence. The Japanese encephalitis virus (JEV) signal sequence from pCBWN [32], [33] and the Kozak sequence [34] were inserted upstream of the prM and NS1 genes to ensure secreted expression of each of these proteins. Sequencing of the expression plasmid confirmed that the recombinant gene fragment was authentic and in frame for correct translation.

COS-7L cells were seeded onto glass cover slips at a density of 2×10^5^ cells per well in 24-well plates overnight (o/n). Transfection was performed using Lipofectamine 2000 (Invitrogen) following manufacturer's instructions. After 21 hr, cells were fixed onto cover slips by soaking in ice cold 100% acetone for 5 min. IFA was performed as described above, however, in this assay, blocking was performed using buffer comprised of 0.05 M Tris–HCl pH 8.0, 1 mM EDTA, 0.15 M NaCl, 0.05%, v/v Tween 20 and 0.2%, w/v casein and the washing was performed with PBS containing 0.05% tween-20 (PBS/T). The bound anti-PCV mAbs were detected with Alexa Fluor 594 goat anti-mouse IgM (Invitrogen).

### PNGase F Digest and Analysis of Secreted Proteins

COS-7L cells were transfected using Lipofectamine 2000 (Invitrogen) following the manufacturer's instructions. As a control for this experiment, recombinant WNV NS1 was produced following transfection of cells with a similar construct to pCBWN [32], [33], except the prM/E genes were replaced with the gene for WNV NS1 (pcBWV-NS1). Cell lysates were harvested at 48 hr post-transfection by adding 150 µl per well (6-well plate) of BS9 lysis buffer (120 mM NaCl, 50 mM H_3_BO_3_, 1% Triton X-100, 0.1% SDS, 5 mM EDTA, pH 9.0) to the cell monolayer. The cells were incubated with lysis buffer on ice for 15 min and then clarified by centrifugation at 12 000 g for 10 min at 4°C and stored at −20°C. For PNGase F digestion, 2 µl of 10% SDS was added to 15 µl of cell lysate and incubated at 95°C for 5 min. After heating, the samples were incubated at 4°C for 2 min before the addition of 1 µl 20% Octyl β-D-glucopyranoside, 2 µl G7 buffer (NEB), and 1 µl PNGase F (NEB). PNGase F digestion was carried out at 37°C for 2 hr, and the samples were analysed by Western blot to determine protein glycosylation indicated by the change in protein mobility as previously described [35].

To assess for secretion of the recombinant NS1 protein, culture supernatant was harvested 48 hr post-transfection and analysed by Western blot. For this Western blot, as well as the PNGase F Western blot, the recombinant PCV proteins were detected using anti-V5 mAb (Invitrogen), while the WNV NS1 protein was detected with the anti-NS1 mAb 4G4 [30].

### Superinfection Exclusion Experiments

Monolayers of C6/36 cells were inoculated at 80% confluency with PCV (M.O.I. ≥1), or sham inoculated with media only, and allowed to incubate for 4–5 days. The cells were seeded into 24 well plates, some containing glass coverslips and incubated for a further 48 hr until cells had almost reached confluency. The growth medium was then removed and cells inoculated with the medically significant flaviviruses Murray Valley encephalitis virus (MVEV) and WNV (Kunjin virus MRM16 subtype – WNV_KUNV_) or the alphavirus Ross River virus (RRV) at an M.O.I. of 0.1 and incubated for 24 or 48 hours. At each time point, the titre of each secondary infecting virus in the culture supernatant was measured by 50% tissue culture infectious dose (TCID_50_) on PS-EK cells and the infectious titre compared with that in sham-infected controls. All samples were tested in triplicate. At each time-point, cell monolayers on coverslips or in wells were fixed in cold acetone. The replication of MVEV, WNV_KUNV_ or RRV was examined by IFA staining using the flavivirus pan-reactive anti-NS1 mAb 4G4 [30] or RRV-specific mAb G8 [36] and imaging performed using the IN CELL Analyzer (GE Healthcare Life Sciences). Co-staining of coverslips was performed by incubation with anti-PCV mAb 3D6 and Alexa-Fluor 488-labelled mAbs 4G4 or G8 (labelled using the Zenon Tricolour Labelling kit (Invitrogen) according to manufacturer's instructions). In this instance, mAb 3D6 was detected with Alexa Fluor 594 goat anti-mouse IgM (Invitrogen). Co-staining images were taken using the ZEISS LSM 510 META confocal microscope.

## Results

### Detection and Isolation of ISFs in Mosquitoes Captured From the Northern Territory

A total of 4194 female mosquitoes as 94 pools were collected from various sites in the Northern Territory of Australia. Mosquitoes of five genera were assessed including *Aedes* (six species), *Culex* (seven species), *Anopheles* (two species), *Coquillettidia* (one species) and *Mansonia* (one species) (data not shown). Just under half (n = 2032) of the mosquitoes tested were *Culex annulirostris*. Thirteen of the pools yielded a PCR product of the expected size upon assessment with the flavivirus RT-PCR. Of these RT-PCR-positive pools, only the *Coquillettidia xanthogaster* mosquitoes yielded flaviviral isolates when inoculated onto C6/36 cultures (Table 1). Partial NS5 gene sequencing revealed that each of these isolates has >98% nucleotide identity to the prototype isolate (isolate 56) and are likely to be a strain of the same virus, which we have tentatively named Palm Creek virus (PCV)(Table 2). Further analysis is being performed on the seven flavivirus isolation-negative pools. ISF-like sequences were amplified from two of these pools and these data will be published in a separate article.

**Table 1:** Summary of PCV isolates from mosquito pools collected in Darwin.

Pool No.	Date Collected	Mosquito Species	Location	RT-PCR*	Isolation#	Virus ID by sequence
22	13 Apr 2010	*Cq. xanthogaster*	Holmes Jungle, Darwin	**+**	**+**	Palm Creek virus (PCV)
56	19 May 2010	*Cq. xanthogaster*	Palm Creek, Darwin	**+**	**+**	PCV
73	8 Jun 2010	*Cq. xanthogaster*	Palm Creek, Darwin	**+**	**+**	PCV
77	4 Jun 2010	*Cq. xanthogaster*	Berrimah Farm, Darwin	**+**	**+**	PCV
90	4 Jun 2010	*Cq. xanthogaster*	Berrimah Farm, Darwin	**+**	**+**	PCV
91	4 Jun 2010	*Cq. xanthogaster*	Berrimah Farm, Darwin	**+**	**+**	PCV

* Mosquito homogenate tested positive in RT-PCR.

# Culture supernatant from inoculated C6/36 cells tested positive in RT-PCR.

**Table 2:** Amino acid and nucleotide identities between PCV and other ISFs over the NS5/3′UTR region.

	PCV56	PCV77	PCV22	PCV73	NAKV	CFAV*	QBV	CTFV	Calbertado	AeFV*	CxFV*	KRV
PCV56	***100***/100	**100**	**100**	**100**	**81**	**57**	**74**	**74.5**	**59.5**	**60**	**70.5–73.5**	**62**
PCV77	99	***100***/100	**100**	**100**	**81**	**57**	**74**	**74.5**	**59.5**	**60**	**70.5–73.5**	**62**
PCV22	98.8	99.6	***100***/100	**100**	**81**	**57**	**74**	**74.5**	**59.5**	**60**	**70.5–73.5**	**62**
PCV73	99.1	99.8	99.5	***100***/100	**81**	**57**	**74**	**74.5**	**59.5**	**60**	**70.5–73.5**	**62**
NAKV	67.6	68.1	68.5	68	***100***/100	**59–60**	**73.5**	**71.5**	**61.5**	**57.5**	**70–73.5**	**60.5**
CFAV	55–56	55.5–56.5	55.3–56.3	55.5–56.5	58.8–59.6	***95.5–100***/94–98	**55.5–56.5**	**54.5–55.5**	**54–55**	**77–78**	**53–56**	**82.5–83**
QBV	66.6	66.6	66.8	66.5	66.1	54.8–55	***100***/100	**85.5**	**59.5**	**57.5**	**83–86.5**	**58.5**
CTFV	65	64.5	64.6	64.5	63.3	53.8–54.3	71.6	***100***/100	**60.5**	**55.5**	**83.5–88**	**57.5**
Calbertado	58.8	59	58.6	59.1	58.3	55.1–55.6	59.3	59.3	***100***/100	**54.5**	**59–61**	**56.5**
AeFV	57.1–57.6	56.6–57.1	56.6–57.1	56.6–57.1	56.8–57.1	67.1–68.3	55.5–55.6	56.8–57.1	53.3–53.5	***100***/98.1–100	**54.5–56.5**	**81**
CxFV	66.1–67.8	66.1–67.8	66.1–67.8	66.1–67.8	63.6–64.8	55.3–58.5	71.6–74	73-74-8	60.1–62.3	55.3–57.8	***92.5–100***/87.3–99.8	**55.5–56.5**
KRV	59.1	58.6	58.8	58.8	59.6	70–71.5	57.5	57	56.8	69.8–70.1	57.5–59	***100***/100

**Bold** text: amino acid identity.

Non bolded text : nucleotide identity.

* A range is given for those viruses that have multiple isolates listed on Genbank.

PCV – Palm Creek virus; NAKV – Nakiwogo virus; CFAV – cell fusing agent virus; QBV – Quang Binh virus; CTFV – Culex theileri flavivirus; AeFV – Aedes flavivirus; CxFv - Culex flavivirus; KRV – Kamiti River virus.

### Sequencing and Phylogeny of the Full Length Genome of PCV

The complete coding region of the PCV genome was sequenced (Genbank accession number KC505248). The PCV ORF encodes a polyprotein that consists of 3,364 amino acids and putative cleavage sites that are similar to those found in the polyproteins of other ISFs (data not shown, [6], [14], [37]). An optimised multiple sequence alignment for PCV and other ISFs over the NS5/3′UTR and ORF regions was performed (Tables 2 and 3). The ORF alignment revealed that PCV is most closely related to the African ISF, NAKV, with 63.7% nucleotide identity (Table 3). Interestingly, the amino acid identity (66.6%) is only slightly higher than the nucleotide identity, suggesting that a large number of these changes are non-synonymous substitutions (Table 3). The next closest relatives of PCV over the entire coding region are QBV, CxFV and CTFV with nucleotide identities of 56.2%, 55.7–56.4% and 55.9–56% respectively. As expected, PCV was also most closely related to NAKV over the NS5/3′UTR region with amino acid and nucleotide identities of 81% and 67.6% respectively.

**Table 3:** Amino acid and nucleotide identities between PCV and other ISFs over the entire coding region.

	PCV56	NAKV	CxFV*	QBV	CTFV*	AeFV	KRV	CFAV*
PCV56	***100/***100	**66.6**	**54–54.4**	**54.5**	**53.8–53.9**	**37.6**	**37.9**	**41.9–42**
NAKV	63.7	***100/***100	**51.8–52.1**	**51.7**	**51.4–51.5**	**37.1**	**38**	**40.3**
CxFV	55.7–56.4	54.5–54.8	***96.3–100***/90.1–100	**69.3–67.7**	**70.7–71.2**	**37.9–38.1**	**38.2–38.4**	**45.3–45.5**
QBV	56.2	54.7	65.8–66	***100***/100	**73–73.1**	**38.3**	**37.6**	**45.5–45.7**
CTFV	55.9–56	54.9	66.8–67	67.6–67.7	***99.3–100***/99–100	**37.6**	**38.5**	**46.5–46.7**
AeFV	44.8	44.5	45.5–45.8	45	45.3	***100***/100	**68.3**	**59.6–60**
KRV	45.3	45.7	45.8–46	45.9	46.2	64.6	***100***/100	**65–65.5**
CFAV	47.3–47.4	47.2–47.4	50–50.4	50.1	50.8	59.4–59.5	62.8–62.9	***97.4–100***/96–100

**Bold** text: amino acid identity.

Non bolded text : nucleotide identity.

* A range is given for those viruses that have multiple isolates listed on Genbank.

PCV – Palm Creek virus; NAKV – Nakiwogo virus; CFAV – cell fusing agent virus; QBV – Quang Binh virus; CTFV – Culex theileri flavivirus; AeFV – Aedes flavivirus; CxFv - Culex flavivirus; KRV – Kamiti River virus.

**Figure 1:**
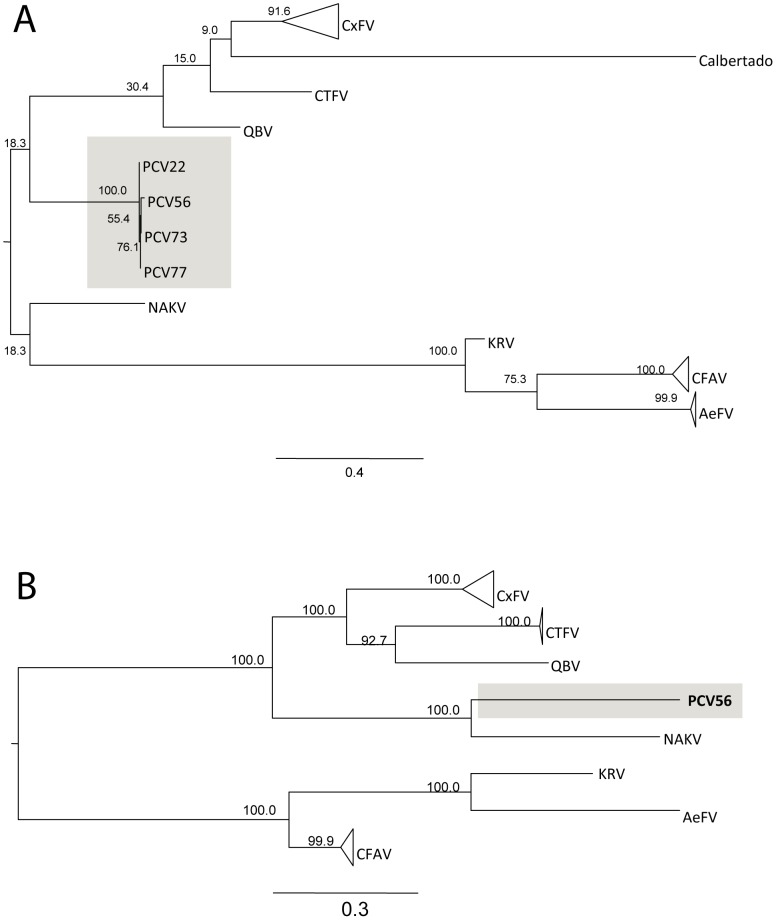
Phylogenetic tree showing relationship between PCV and other insect-specific flaviviruses. (A) Maximum likelihood tree constructed using NS5/3′UTR sequences of select insect-specific flaviviruses and four isolates of PCV. (B) Maximum likelihood tree constructed using the complete ORFs of a selection of insect-specific flaviviruses. For both trees, the numbers at the nodes represent bootstrap replicates as a percentage of 1000 replicates. Both trees have been mid-point rooted. CFAV and CxFV groups have been collapsed for clarity.

Phylogenetic analysis of the nucleotide sequence of the NS5 and partial 3′ UTR regions of four PCV isolates revealed that these isolates are highly similar and cluster with other ISFs, although the bootstrap support is low, and we were unable to conclude the location of PCV in the ISF group from this region alone (Fig. 1A). In contrast, bootstrap support for the complete genome tree is high and shows that PCV clusters most closely with NAKV virus, with 100% bootstrap support (Fig. 1B).

### TEM Analysis of Virus Particle Morphology

**Figure 2:**
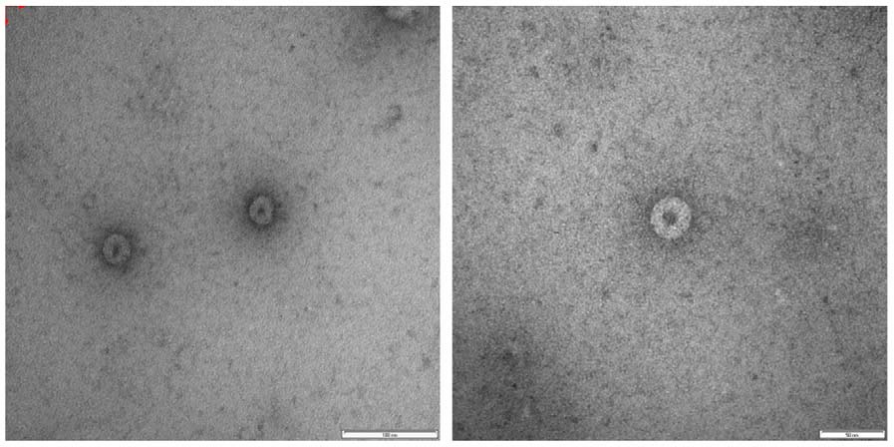
Transmission Electron Micrograph of PCV particles. Culture supernatant from C6/36 cells infected with PCV was concentrated and round, enveloped virus particles with an electron-dense core were visualised using uranyl acetate staining. Scale bars are 100 nm and 50 nm for left and right panels respectively.

When concentrated culture supernatants from PCV-infected C6/36 cell cultures were examined under TEM, small spherical particles about 40–50 nm in size were observed (Fig. 2), consistent with the expected size of flavivirus particles [38].

### Growth of Viral Isolates in Different Cell Lines

The ability of PCV to infect a range of cells was assessed by regularly monitoring the cell monolayers for evidence of CPE and by assaying the cell monolayers for the presence of viral RNA by RT-PCR. While PCV replicated in C6/36 cells, as determined by a positive reaction in RT-PCR, no evidence of growth was observed in any of the vertebrate cells lines tested (Table 4). Although the first and second passage of each isolate showed no evidence of CPE in C6/36 cells, often by passage 4 there was clear evidence of morphological changes such as syncytia and vacuolation in cells infected with each isolate (Fig. S1B). It was also observed that fusion of the C6/36 cells by PCV could be enhanced by reducing the pH of the cell culture medium to pH 6 (Fig. S1D and E). These observations are consistent with previous studies with this cell line and other flaviviruses [39].

**Table 4:** Replication of PCV56 in various cell lines.

Isolate	Replication of Viral Isolates in various cell lines									
	C6/36		BHK-21		PS-EK		Vero		SW13	
	CPE	RT-PCR	CPE	RT-PCR	CPE	RT-PCR	CPE	RT-PCR	CPE	RT-PCR
PCV56	***+***	***+***	**−**	**−**	**−**	**−**	**−**	**−**	**−**	**−**
WNV_KUNV_ *	***+***	ND	**+**	ND	***+***	ND	***+***	ND	**+**	ND

* KUNV used as positive control in these experiments.

ND Not done.

### Monoclonal Antibodies to PCV-Specific Antigens

Our initial analysis of the PCV isolates with a panel of mAbs that were pan-reactive to vertebrate-infecting flaviviruses (e.g. mAb 4G2 [40] and mAb 4G4 [30]), revealed a lack of recognition in a fixed-cell ELISA and Western blot (results not shown) and confirms that these viruses are antigenically distinct from other members of the flavivirus genus.

**Figure 3:**
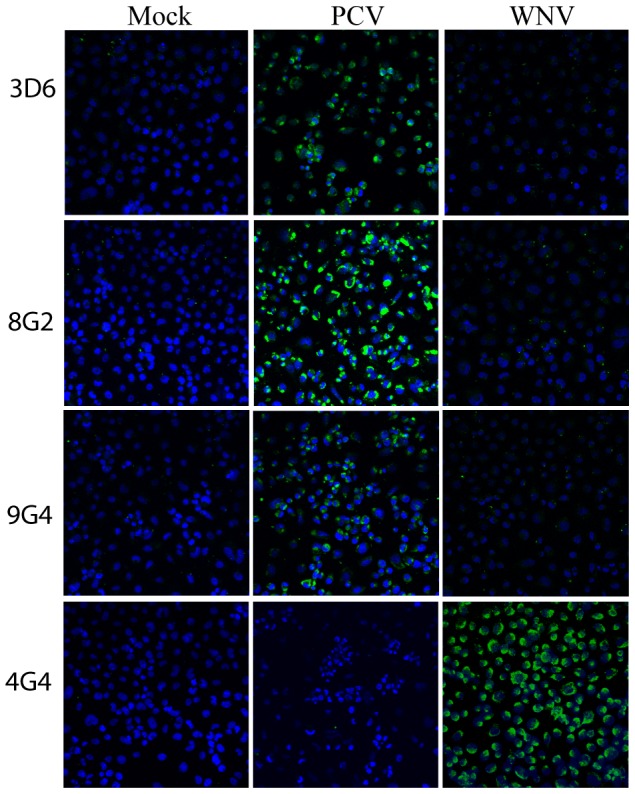
Reactivity of anti-PCV mAbs to PCV-infected C6/36 cells by IFA. Mock-, PCV and WNV_KUNV_-infected C6/36 cell monolayers were fixed using acetone and probed with anti-PCV mAbs 3D6, 8G2 and 9G4 or with 4G4 which binds to WNV_KUNV_ NS1 protein [30]. The nucleus of each cell was stained by Hoechst. Images were taken using a ×40 objective lens.

To obtain antibodies reactive to PCV antigens as research tools for further study of this virus and possibly other ISFs, hybridomas were produced to a preparation of concentrated PCV virions from infected mosquito cell culture supernatant. Three mAbs (3D6, 8G2, 9G4) that recognise PCV-specific antigens were obtained from the resulting hybridomas. These mAbs reacted with acetone-fixed PCV-infected cells by IFA and ELISA and failed to bind to uninfected cells in these assays (Fig. 3). As expected, no reaction was detected to cells infected with any of the vertebrate-infecting flaviviruses including WNV_KUNV_, MVEV, yellow fever virus (YFV) and dengue virus (DENV), consistent with our earlier data (Table 5).

**Table 5:** Characterisation of anti-PCV mAbs.

mAb ID	Isotype	Protein specificity	Reactivity in WB	Virus Specificity*				
				PCV	WNV	MVEV	DENV	YFV
3D6	IgM/*_K_*	NS1	−	+	−	−	−	−
8G2	IgM/*_K_*	?	−	+	−	−	−	−
9G4	IgM/*_K_*	NS1	−	+	−	−	−	−

* Binding pattern of mAbs in fixed cell ELISA to PCV, Palm Creek virus; WNV, West Nile virus Kunjin subtype; MVEV, Murray Valley encephalitis virus; DENV, Dengue virus, YFV, yellow fever virus.

WB = Western blot.

The three anti-PCV mAbs were all of the IgM isotype and recognised highly conformational epitopes that were sensitive to SDS-denaturation and hence, did not react with PCV antigens in Western blot (Table 5).

### Recognition of Recombinantly Expressed PCV NS1 Protein by Anti-PCV mAbs

**Figure 4:**
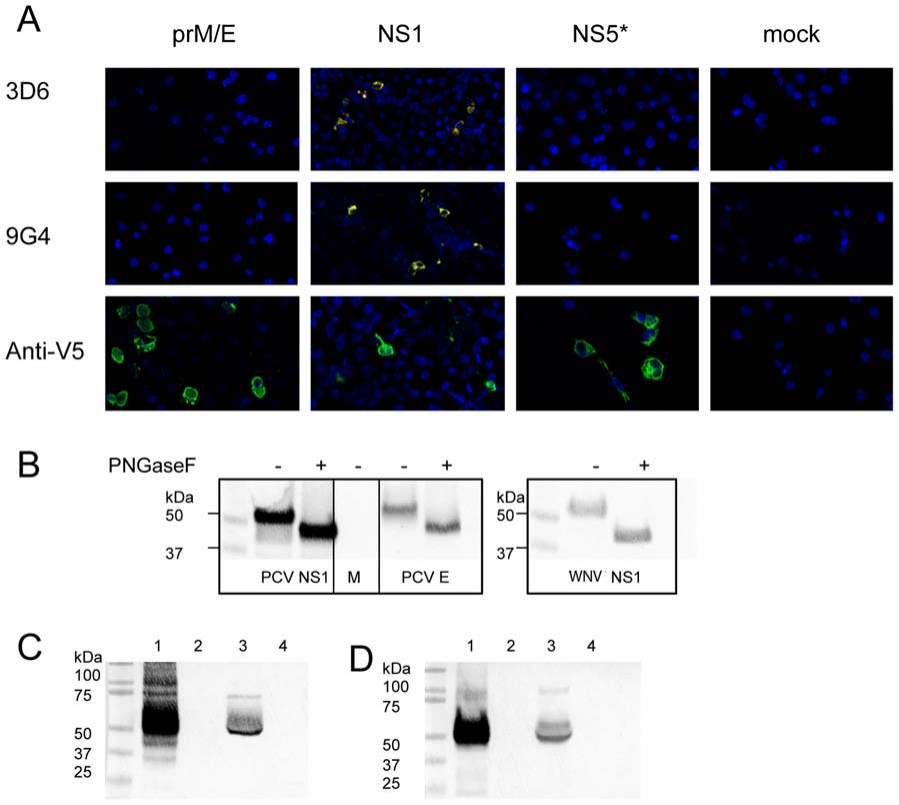
Analysis of recombinant PCV proteins. COS-7L cells were transiently transfected with pcDNA3.1-based plasmids encoding genes for the expression of PCV secreted prM/E, NS1 and partial NS5. Each expressed protein contained a C-terminal V5 tag. Mock cells were transfected with the empty pcDNA3.1 construct. **A**) Cells were fixed 21 hr post-transfection and probed with mAbs 3D6, 9G4 or anti-V5 using IFA. **B**) Lysates of COS-7L cells transfected with plasmids encoding PCV NS1, PCV secreted prM/E, WNV NS1 or pcDNA3.1 (M = mock) were treated (+) or untreated (−) with PNGaseF to remove N-linked glycans. The proteins were probed with anti-V5 (left panel) or anti-NS1 mAb 4G4 (right panel) in Western blot. **C,D**) Western blot analysis of supernatant and lysate of transiently transfected cells with PCV NS1 construct (**C**) or WNV NS1 construct (**D**). Protein detection was with anti-V5 and mAb 4G4 respectively. Lane 1 – NS1 lysate; Lane 2 – mock lysate; Lane 3 – NS1 supernatant; Lane 4 – mock supernatant. *Partial NS5 expressed. The construct is missing the sequence for the first 85 amino acids of the predicted full length PCV NS5 protein.

To assist in determining which PCV viral protein is recognised by each of the anti-PCV mAbs, three constructs were prepared for the expression of partial NS5 and the secreted expression of NS1, prM and E proteins. Each gene was cloned into a mammalian expression vector, upstream of the genes for V5 and HIS affinity tags. COS-7L cells were transiently transfected with each of these constructs and when the cells were fixed post-transfection, expression of recombinant protein was confirmed by the specific reaction of the anti-V5 antibody to the affinity tag fused to the expressed protein in IFA (Fig. 4A). Furthermore, two of the anti-PCV mAbs (3D6 and 9G4) specifically recognised the recombinant NS1 protein expressed in the transfected cells. The third anti-PCV mAb (8G2) did not recognise any of the recombinant proteins (data not shown).

### Analysis of Recombinant PCV NS1 and E Proteins

To confirm whether the PCV E and NS1 proteins were glycosylated, the recombinantly expressed proteins were digested with PNGase F to remove N-linked glycans. Recombinant WNV NS1, which expresses three N-linked glycans, was similarly digested. Removal of the N-linked glycans is characterised by an increase in the mobility of the proteins and was seen for both PCV E and NS1 proteins to a similar extent as WNV NS1 (Fig. 4B). These data suggests that both PCV E and NS1 proteins are likely to utilize two to three glycosylation sites. This is consistent with the *in silico* predicted glycosylation sites for PCV NS1 and E proteins (two and three sites respectively; Net Nglyc 1.0 Server, www.cbs.dtu.dk/services/NetNGlyc).

Secretion of recombinant PCV NS1 protein was confirmed following the harvesting of the transfection cell culture supernatant and detection of the expressed protein in Western blot (Fig. 4C). While the NS1 monomer was clearly detected at a molecular weight of approximately 50 kDa, a fainter band with an apparent molecular weight of approximately 70 kDa was also visible. While it could be suggested that this larger band is an NS1 dimer, it is considerably smaller then the corresponding band for recombinant WNV NS1 dimer which is visible at approximately 100 kDa (Fig. 4D).

### PCV Suppresses the Replication of Medically Significant Flaviviruses *In Vitro*

**Figure 5:**
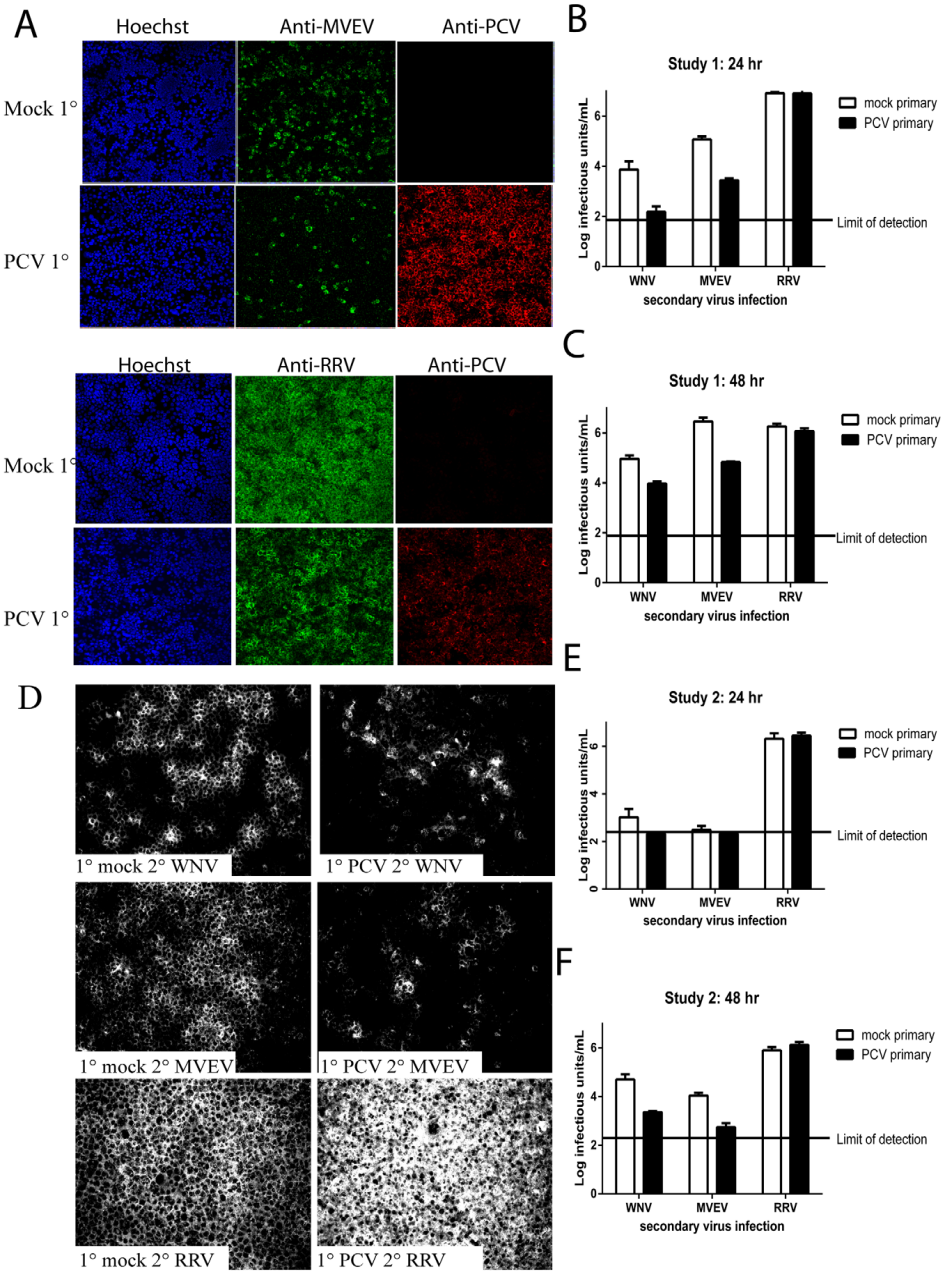
*In vitro* superinfection exclusion assay. IFA and infectious titre assays showing reduced replication of the flaviviruses MVEV and WNV_KUNV_ in C6/36 cells persistently infected with PCV when compared to mock infected (PCV-negative) cells. The alphavirus RRV replicates equally well in both. A–C Study 1; D–F Study 2. A) Co-staining was performed to detect the primary and secondary infecting viruses. The secondary viruses MVEV and RRV were detected with Alexa Fluor 488-labelled mAbs 4G4 and G8 respectively. The PCV primary infection was detected with the anti-PCV mAb 3D6. D) Dramatically less staining for the secondary infecting viruses WNV_KUNV_ and MVEV was detected with mAb 4G4 in cells that were persistently infected with PCV compared with mock-infected cells. In comparison, RRV grew equally well in PCV- and mock-infected cells, as detected with mAb G8.

To determine whether prior infection of mosquito cells with ISFs could suppress subsequent infection with pathogenic, vertebrate-infecting flaviviruses, C6/36 cultures were infected with PCV (or they were mock-infected) 6–7 days prior to a secondary inoculation with the flaviviruses, MVEV or WNV_KUNV_. The infectious titres of the secondary infecting virus was compared to those produced in the PCV-negative controls. The results show that the infectious titres of the secondary infecting flaviviruses in study 1 were significantly reduced in PCV infected cells compared to PCV-negative cells at 24 hr (WNV_KUNV_ 1.7 log reduction, *p* = 0.012; MVEV 1.63 log reduction, *p* = 0.0003 Student's two-tailed t test) (Fig. 5B). A reduction in the infectious titre of WNV_KUNV_ and MVEV in PCV-infected cells was similarly observed at 48 hr (Student's two-tailed t test WNV_KUNV_ *p* = 0.0032 (1 log), MVEV *p* = 0.0004 (1.63 log)) (Fig. 5C). This was consistent with the IFA images which showed dramatically reduced numbers of infected cells in the primary infected cultures compared to the mock-infected controls (Figs. 5A and D). In comparison, infection with the alphavirus RRV (which uses a different replication strategy) was permissive to the same level in both PCV-infected and uninfected cells (Student's two-tailed t test *p*>0.05, Figs. 5B and C) confirming the flavivirus-specific nature of the replication suppression in PCV-infected cells and demonstrating that cells persistently infected with PCV were sufficiently healthy to support replication of an unrelated positive strand RNA virus to the same extent as uninfected cells. These data were confirmed in a separate experiment where a similar trend in reduction of the infectious titres of WNV_KUNV_ and MVEV in PCV-infected cells in comparison to mock infected cells was observed (Figs. 5E & F). Although at 24 hr the infectious titres of MVEV were below the limit of detection (Fig. 5E), by 48 hr a significant reduction in the infectious titres of both WNV_KUNV_ and MVEV in PCV-infected cells was observed (Student's two-tailed t test WNV_KUNV_ *p* = 0.0037 (1.34 log), MVEV *p* = 0.0031 (1.3 log)). Together, these data represent the first demonstration that mosquito cells persistently infected with an ISF are considerably less permissive to pathogenic flaviviruses 24–48 hr post-infection.

## Discussion

The discovery of PCV in mosquito populations in Darwin represents the first isolation of an ISF in Australia. Our phylogenetic data also reveal a close relationship between PCV and other ISFs, placing them in the same clade as the prototype ISF (CFAV) and most of the more recent ISF isolates from around the world [4], [6], [8]–[10], [14], [23], [41]. Isolation of PCV from *Coquillettidia xanthogaster* also represents the first isolation of an ISF from a member of the *Coquillettidia* genus.

Presently, the distribution of PCV and its insect host restriction in Australia is not clear; however a preliminary study of mosquitoes trapped in the Kimberley region of north-western Australia have detected PCV-like viruses in both *Coquillettidia* and *Culex* species suggesting the virus could be more widespread and present in additional mosquito species (Nguyen, McLean, Hobson-Peters, Barnard, Johansen and Hall, unpublished data). Indeed, in this present study, PCV was isolated from 33.3% of the *Cq. xanthogaster* pools assessed and suggests a high prevalence of this ISF in *Coquillettidia* mosquito populations within the Northern Territory of Australia.

Our phylogenetic data suggests that PCV is most closely related to NAKV, an ISF isolated from *Mansonia* species in Uganda. However, these viruses share only 63.7% nucleotide identity over the ORF, which clearly indicates that PCV represents a new species [22] and that PCV and NAKV have clearly evolved separately for some time. It is worth noting that *Mansonia* mosquitoes are closely related to *Coquillettidia*, both belonging to the Mansoniini mosquito tribe [42]. This is suggestive of co-evolution of these viruses with their mosquito hosts. However this hypothesis is not supported by the work of Cook et al. (2012) where no significant proof for the co-divergence of insect-specific flaviviruses and their vectors was identified [43].

Our *in vitro* findings that mosquito cells previously infected with PCV were less permissive to subsequent infection with WNV_KUNV_ and MVEV, in a flavivirus-specific manner, provide evidence that ISFs may down-regulate the replication of vertebrate-infecting flaviviruses in mosquito tissues. However, *in vivo* studies are yet to be performed and it must be noted that the response to flaviviral infection *in vivo* may differ to that seen in C6/36 cells due to the defective innate immune response of these cells [44], [45]. Despite this, several *in vitro* studies have shown that prior infection of cells with one flavivirus can inhibit replication with a related flavivirus subsequently inoculated into the culture [46]–[48]. This phenomenon is known as superinfection exclusion and has been postulated as a mode of competition for mosquito hosts between related viruses. The molecular mechanisms of superinfection exclusion are thought to involve competition for, or modification of cellular factors that result in reduced receptor binding, viral entry or RNA replication [49]. Indeed, recent studies by Zou et al, (2009) using WNV and mammalian cells (BHK-21), identified a mechanism of superinfection exclusion between flaviviruses at the step of viral RNA synthesis, reportedly due the sequestering of cellular factors (host proteins and membranes) to support RNA replication of the primary infecting virus, thereby depriving the second infecting virus of essential elements of replication [50].

Interactions between CxFV and WNV have been assessed *in vitro* by other groups. In one similar experiment in C6/36 cells, primary infection with CxFv and subsequent infection with WNV 48 hr later resulted in significant differences in the WNV titres, but not until 84–156 hr post-infection [17]. This contrasts our data where we observed differences in the titres of WNV_KUNV_ and MVEV at 24 and 48 hr post-infection. In another study, C6/36 cells sequentially infected with CxFv and WNV also resulted in lower titres of infectious WNV secreted from cells infected with CxFV compared with those that were mock infected. However, these differences were not statistically significant and death of the cells infected with CxFv was also observed [18].

The high prevalence of ISFs in some populations of mosquitoes has prompted speculation on the role of ISFs in regulation of the transmission of pathogenic flavivirus by superinfection exclusion or similar mechanism. However, recent studies report that co-infection of *Cx. quinquefasciatus* mosquitoes from Honduras with an ISF from Guatemala (CxFV Izabal) and WNV significantly enhanced the transmission rate of the latter [18]. This was consistent with reports by Newman et al. (2011) of a positive ecological association between CxFV and WNV in co-infected mosquito pools collected in the field, whereby pools of *Cx. pipiens* that were positive for WNV, were four times more likely to also be positive for CxFV [51]. Competitive interaction between CxFV and WNV *in vivo* has also been observed by Bolling et al. (2012) [17]. In this vector competence study using *Cx. pipiens* mosquitoes, early suppression of WNV replication and dissemination was seen in mosquitoes persistently infected with CxFV. While the mechanism is not yet understood, these preliminary studies provide the first evidence that ISFs may affect the transmission of pathogenic mosquito-borne viruses in nature. However, the large genetic diversity between ISFs identified in different parts of the world and our own data showing an inhibitory effect of PCV on the replication of other Australian flaviviruses *in vitro*, indicates that this relationship may vary with the species of virus (and mosquito) under examination. *In vivo* experiments are currently underway to determine whether prior infection of Australian mosquito species with PCV can also inhibit or delay the transmission of WNV_KUNV_.

The derivation of three hybridomas reactive to PCV antigens in this study is the first report of mAbs specific for an ISF. The lack of growth of these viruses in vertebrate cells and the absence of consistent and clearly observable CPE that they produce in mosquito cells, particularly at low passage, has to date required the use of RT-PCR to detect and monitor the growth of these viruses in mosquitoes and cell culture. Two of the mAbs were shown to be reactive to the PCV NS1 protein. Since the epitope recognised by these mAbs is sensitive to SDS denaturation, it is possible that these mAbs specifically bind a larger NS1 dimeric or hexameric complex [52] and would be consistent with the method of immunogen concentration from cell culture supernatant using large molecular weight cut off concentrators. The protein bound by the third mAb (8G2) could not be determined. The epitope bound by this mAb may not be authentically expressed on the recombinant proteins, or, this mAb may bind E or prM/E complexes. Although recombinant prM/E was assessed in this study, it should be noted that the lack of the E protein transmembrane domain, as well as the presence of the C-terminal V5/HIS tag is likely to have prevented sub-viral particle (SVP) assembly. A construct for the expression of PCV SVPs is currently being made.

The three mAbs appear to be specific for PCV as no cross-reactivity was seen to any of the vertebrate-infecting flaviviruses assessed and our preliminary data suggests that there is negligible or no reactivity to other ISFs. This provides evidence that PCV is antigenetically distinct from other closely related ISFs, although further assessment of these mAbs with PCV's closest relative, NAKV is required.

Digestion of the recombinant PCV NS1 and E proteins with PNGase F revealed that both of these proteins possess two or three N-linked glycans. While the E protein of the vertebrate-infecting flaviviruses, such as WNV and MVEV is normally singly glycosylated, or not glycosylated at all [35], [53]–[55], the insect-specific flaviviruses QBV and KRV contain 6 potential E glycosylation sites, although only 2–3 are utilised [4], [9].

In summary, we provide the first isolation of an ISF from mosquitoes collected in Australia. Additionally, we report the first ISF-specific mAbs. The advent of these new reagents will allow rapid detection of PCV *in vitro* and *in vivo* and will be useful tool for further research on the biology of these viruses. A thorough investigation on the ecology and epidemiology of this virus and of similar viruses circulating in Australia will provide valuable insight into the biological relevance of this important group of viruses.

## Supporting Information

Figure S1 **Phase contrast microscopy of C6/36 cells infected with PCV at pH 6 and pH 7.** Mock (A) and PCV-infected cells (B) four days post-infection with virus at passage 4 under standard culturing conditions. Fusion of the PCV-infected cells was enhanced by reducing the culture medium pH to 6: (C) Uninfected C6/36 cells (×200) in pH 6 medium; (D) PCV-infected cells (×200) in pH 6 medium; (E) PCV-infected cells (×400) in pH 6 medium; (F) PCV-infected cells (×400) in pH 7 medium.
(TIF)
