# Supplementary figures and images for: A New Insect-Specific Flavivirus from Northern Australia Suppresses Replication of West Nile Virus and Murray Valley Encephalitis Virus in Co-infected Mosquito Cells
Source: PLoS One. 2013 Feb 27;8(2):e56534. doi: 10.1371/journal.pone.0056534 (PMC3584062; doi:10.1371/journal.pone.0056534)

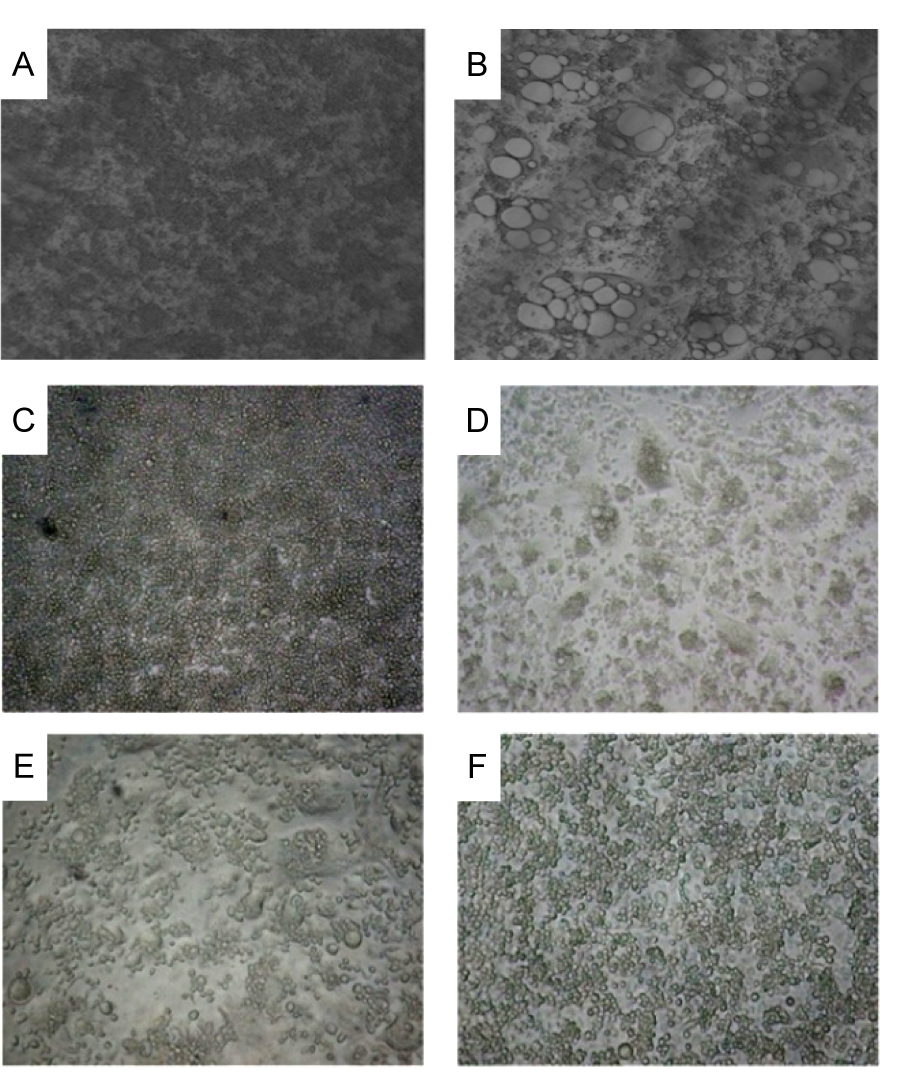

Supplement: Figure S1 — Phase contrast microscopy of C6/36 cells infected with PCV at pH 6 and pH 7. Mock (A) and PCV-infected cells (B) four days post-infection with virus at passage 4 under standard culturing conditions. Fusion of the PCV-infected cells was enhanced by reducing the culture medium pH to 6: (C) Uninfected C6/36 cells (×200) in pH 6 medium; (D) PCV-infected cells (×200) in pH 6 medium; (E) PCV-infected cells (×400) in pH 6 medium; (F) PCV-infected cells (×400) in pH 7 medium. (TIF) [file pone.0056534.s001.tif]
